# General practice management of chronic post-surgical pain in patients with hip fracture: a qualitative study

**DOI:** 10.3389/fmed.2023.1304182

**Published:** 2024-01-15

**Authors:** Wenshu Cao, Jizhong Ye, Yini Yan, Cheng Xu, Qiwei Lv

**Affiliations:** ^1^Tianlin Community Health Center of Xuhui District, Shanghai, China; ^2^Department of Anaesthesiology, Shanghai Sixth People's Hospital Affiliated to Shanghai Jiao Tong University, Shanghai, China

**Keywords:** hip fracture, chronic post-surgical pain, general practitioners, evidence-based practice, primary care

## Abstract

**Background:**

Hip fractures are common among elderly people and often lead to chronic post-surgical pain (CPSP). Effective CPSP management when patients transition from hospital to community settings is essential, but has not been sufficiently researched. This study examined general practitioner (GP) perspectives on managing patients with CPSP after hip fractures in Shanghai, China.

**Methods:**

A descriptive qualitative study was performed wherein semi-structured interviews were conducted with GPs practicing in Shanghai who volunteered to participate. This study was initiated after a regional survey of general practice care for patients with CPSP following hip fracture.

**Results:**

Six key themes emerged: (1) GPs’ care priorities for patients with CPSP varied; (2) pharmacological management posed challenges in terms of selecting appropriate medications; (3) consultation time constraints hindered comprehensive management; (4) GPs desired better communication from hospitals at discharge; (5) limited access to services, such as pain specialists and allied health, obstructed optimal care delivery; and (6) patient nonadherence to CPSP treatment was an issue.

**Conclusion:**

Multiple patient-, provider-, and system-level factors affected GP care for patients with CPSP after hip fracture. Improved interdisciplinary communication and education on evidence-based CPSP guidelines are needed to address the knowledge gaps among GPs. Barriers to healthcare access must be minimized to facilitate guideline-based care.

## 1 Introduction

Osteoporotic hip fractures (HF) are a prevalent cause of morbidity and mortality in the elderly population (1). HF frequently occurs as a consequence of a traumatic fall. In 1999, HF affected 338,000 people aged 65 years and older, and this number is projected to surpass 500,000 by 2040 (2). In 2016, the age-standardized incidence of HFs in the urban areas of China was 177 per 100,000 women, and 99 per 100,000 men. However, the total number of hip fractures in people aged 55 years and older increased approximately four-fold between 2012 and 2016, owing to population aging (3). Following HF surgery, patients commonly experience pain near the fracture and wound, which typically subsides as bone fusion and wound healing occur. Nevertheless, pain may persist despite the progression of healing (4). According to previous reports, a subset of patients with HF experience chronic persistent pain 6 months after surgical intervention. This condition is closely linked to disability (5, 6).

Perioperative management of HF presents a complex challenge for patients and healthcare providers. Pain is a prevailing concern, and optimal analgesia is not achieved consistently (7). Pain induces substantial distress in patients and gives rise to well-documented physiological consequences including tachycardia, hypertension, and heightened cardiac workload, which may endanger patients with cardiac comorbidities (8). Effective pain management is crucial for mitigating the onset of chronic post-surgical pain. Furthermore, extensive studies have examined the correlation between the severity of acute postoperative pain and the likelihood of transitioning to persistent pain (9, 10).

Chronic post-surgical pain (CPSP) can occur after various surgical procedures and may persist for >3 months after surgery. However, certain surgeries carry a higher risk of CPSP owing to extensive tissue damage, significant inflammatory response, or nerve injury (11, 12). Numerous studies have established a correlation between CPSP and surgeries involving HF and hip replacement (4, 13–15). However, very few investigations on the occurrence of CPSP among patients who have undergone previous HF repair have been conducted (16). A considerable portion of the available literature primarily focuses on hospital-centered strategies for managing pain, particularly multimodal interventions that encompass analgesic drugs (e.g., opioids, nonsteroidal anti-inflammatory drugs, and acetaminophen) and technology bundles (e.g., peripheral nerve blockade). The main objective of these interventions is to reduce the intensity of acute pain (17). Moreover, the current literature has not yielded empirical evidence regarding the impact of any intervention on the development of chronic pain in this specific group of patients undergoing surgery.

A significant concern that remains unexplored pertains to the efficacious transfer of care from acute settings to community-based primary care. With the depth of China’s national healthcare reform, the health sector has increased redistribution at the systemic level and between different coordinating agencies, allowing progress in universal health coverage (UHC) in primary care. In Shanghai, China, where the present study was conducted, general practitioners (GPs) are suitable candidates for effective CPSP prevention and follow-up treatment in patients with HF. They are well positioned to provide comprehensive CPSP care for patients with HF in a UHC-based setting, as well as continuity, coordination, and integration of care with other health professionals and services. Although CPSP is a prominent contributor to avoidable hospital admissions, there has been limited assessment of care provision and adherence to evidence-based guidelines in general practice settings.

Therefore, this study aimed to investigate GP perceptions regarding CPSP management in patients with HF and elucidate the factors influencing the provision of evidence-based care in public health settings in Shanghai, China.

## 2 Methods

### 2.1 Trial design and participants

A qualitative descriptive study was conducted to examine the perspectives of GPs in Shanghai on the management of CPSP following HF. Participants were public primary care physicians working in geographic locations with similar socioeconomic statuses within the Shanghai urban area. As one of the most important cities in China, Shanghai incorporates advanced primary healthcare management concepts and medical technology. The treatment philosophy of GPs in public community hospitals is relatively cutting edge and representative of the general GP population. The interviews were guided by established principles for CPSP management and feedback from GPs in the Shanghai Region survey, which focused on providing general practice care to patients experiencing CPSP after undergoing HF surgery. The study data adhered to the Standards for Reporting Qualitative Research (SRQR) (18) guidelines. Submission to an ethics committee for approval was not required as this study primarily constitutes an opinion survey.

### 2.2 Recruitment

Participation was sought from GPs in the Shanghai region who had completed a regional survey on GP care for patients recovering from CPSP after HF surgery. The survey was purposively distributed to practice GPs across all territories in Shanghai who had provided care for such patients within the past 2 years. Subsequently, the participants voluntarily expressed their interest in participating in a qualitative interview to delve deeper into their experiences and to provide improved care for this patient population. This approach can be considered as a convenience sample for the interview process.

### 2.3 Data collection

Between July and December 2022, the lead author, a practicing GP, conducted interviews using the Tencent video conference platform. The interviews were scheduled at the convenience of the participants. To guide the interviews, a semi-structured interview guide was developed based on the approach outlined by Minichello et al. (19), which employs a recursive model of interviewing, utilizing a combination of closed and open-ended questions. The interviews aimed to investigate GP perceptions regarding CPSP management in patients with HF and the factors influencing the provision of evidence-based care following guide recommendations (Table 1). During the interviews, the participants were actively prompted to express their perspectives and encounters regarding providing care to patients experiencing CPSP following HF surgery. The interviews were meticulously transcribed in their entirety by a proficient transcriptionist specializing in digital recordings. Field notes and a reflective research journal were maintained to establish an audit trail and facilitate reflexive thematic analysis. All interviews were transcribed verbatim and translated from Chinese to English, and two assistants coded each transcript independently.

**Table 1:** One illustration of open-ended inquiries posed within the framework of the semi-structured interview protocol.

Number	Questions		
No. 1	What is the perceived significance of follow-up appointments with GPs for patients experiencing CPSP following Hip fracture surgery?		
No. 2	What are the significant concerns that you would like to have addressed during your subsequent appointment following hip fracture surgery in the event of CPSP?		
No. 3	What is your experience with CPSP in hip fracture patients transitioning from hospital to community care?		
No. 4	What are the obstacles encountered in the provision of evidence-based care for these patients?		
No. 5	What strategies would you propose for the prevention and enhancement of care for postoperative hip fracture patients who are admitted to the hospital due to CPSP?		

CPSP, chronic postsurgical pain; GP, general practitioner.

After the initial three interviews, the authors thoroughly examined the transcripts and the interviewers’ reflections. This process aimed to enhance the interview guide and offer constructive criticism of the interview techniques. Additionally, all participants were compensated for any clinical time lost with a gift voucher, and were offered the opportunity to review their unedited transcripts to clarify or elaborate on their initial responses. It is worth noting that repeated interviews were deemed unnecessary, although two participants did return their transcripts with minor modifications. The recruitment phase concluded after a total of 17 interviews. At this point, the interviews yielded no novel concepts, leading us to conclude that the sample size was adequate to address the study’s objectives.

### 2.4 Data analysis

The qualitative data management and analysis software, NVivo 12, was employed to facilitate coding and analysis. The first author extensively reviewed the transcripts multiple times before coding and thematic analysis. This analysis followed the 6-step approach to reflexive thematic analysis outlined by Ayre and McCaffery (20), which includes familiarizing oneself with the data, generating initial codes, searching for themes, reviewing themes, defining and naming themes, and ultimately producing the final report. The first author developed a preliminary coding scheme after conducting an initial review of the transcripts. Subsequently, the co-authors engaged in further discussion, questioning, and probing, leading to refinement of the initial coding scheme. Once finalized, the coding scheme was applied to the entire set of transcripts. A thematic map and charting were employed across the dataset to explore and comprehend the relationships between concepts and to identify and clarify patterns and features based on meaning. In addition, we applied vote resolution to handle conflicts, considering both average coverage and consensus sequence length. Multiple meetings were conducted to enhance and specify candidate themes and elucidate the extent and fundamental aspects of each theme before confirming them.

## 3 Results

### 3.1 Study participants

A total of 25 GPs initially indicated their willingness to participate in the qualitative interviews. However, after this expression of interest, three GPs withdrew their participation, whereas five others failed to respond to email or telephone correspondence. Ultimately, a cohort of 17 participants (seven males and 10 females) with diverse experience in general practice spanning 3 to 29 years and covering various regions in Shanghai, were included in the study (as depicted in Table 2). The average interview duration was 20 min, ranging from 15 to 38 min.

**Table 2:** Participant characteristics.

Variable	Number of participants (%)
Gender	
Male	7(41.2)
Female	10(58.8)
Years of experience	
1–5	5(29.4)
6–20	7(41.2)
>20	5(29.4)
Area of affiliation	
Xuhui district of Shanghai	4(23.5)
Minhang district of Shanghai	5(29.4)
Huangpu district of Shanghai	4(23.5)
Putuo district of Shanghai	4(23.5)

Analysis of the interview data revealed six prominent themes that influenced the provision of GP care to patients with chronic pain resulting from hip fractures.

***Theme 1. The priorities of GPs regarding care for HF patients in the context of CPSP exhibited a range of*** var ***iations.***

A consensus was reached among all participants regarding the significance of scheduling a postoperative follow-up appointment with a GP for patients with hip fracture and CPSP. However, divergent opinions emerged concerning the prioritization, agenda, and timing of follow-up appointments. A subset of GPs advocated for a primary focus on assessing the patient’s pain level and reviewing medications during these visits.

*“It is crucial to assist patients in assessing whether their condition necessitates analgesic intervention and to ensure adherence to prescribed analgesic medication.”* (GP 10, with 10 years of experience).

Many GPs possess a comprehensive understanding of CPSP and regard its prevention and pain management during readmission as paramount concerns.

*“Inadequate management of a CPSP increases the likelihood of recurring pain.”* (GP 02, with 9 years of experience).

*“We have implemented a comprehensive management plan for postoperative CPSP in patients with hip fracture, which encompasses optimal acute pain management strategies. This approach aligns with current evidence-based best practices for preventing and treating postoperative CPSP.”* (GP 05, with 3 years of experience).

GPs perceive the follow-up of CPSP after HF as a valuable opportunity for healthcare professionals to reassess their treatment strategies and enhance patient care to mitigate the risk of future pain exacerbations. Moreover, GPs believe that their enduring doctor-patient relationship placed them in an advantageous position to deliver preventive measures.

*“It is incumbent upon us to acknowledge CPSP as a potential risk factor for patients undergoing hip fracture surgery. Given our comprehensive understanding of the patient’s condition, we are responsible for formulating a strategic approach to prevent further decline in their health status.”* (GP 03, with 5 years of experience).

A limited number of GPs in this study indicated the use of treatment guidelines for managing CPSP after HF. Among the GPs who knew the Chinese guidelines for chronic pain management, their perspectives exhibited diversity. Some expressed that the guidelines were excessively general or that they encountered difficulties accessing the required information. Conversely, others deemed the guidelines to lack utility, while a separate group perceived them as less intricate and more user-friendly. Experienced GPs did not dismiss the guidelines outright but instead favored an individualized approach to practice. Nevertheless, they acknowledged the need to enhance their knowledge to align their practices with the established guidelines.

Implementing action plans to manage CPSP exacerbations after HF is considered a crucial component. Several GPs perceived these plans as highly beneficial in effectively mitigating exacerbations and averting hospitalization.

*“The majority of my patients possess well-defined action plans to mitigate the occurrence of persistent postoperative pain. I have consistently encountered no issues in this regard, as my patients are sincerely committed to adhering to the provided guidance. Ensuring that the action plan is uncomplicated and comprehensible is imperative. Consequently, only a minimal proportion of my patients who have undergone hip fracture surgery necessitate hospital re-admission for pain control.”*

On the contrary, specific GPs held a less favorable perspective on action plans, positing that elderly patients experiencing CPSP were uninterested in receiving “an additional piece of documentation.” Several GPs preferred providing verbal guidance over written plans. Nevertheless, the GPs acknowledged that their involvement in this study prompted them to incorporate written action plans more extensively into their future clinical practice to manage CPSP following HF. Participants believed that the implementation of action plans was hindered by barriers such as inadequate consultation time and limited health literacy.

***Theme 2. The management of CPSP in patients following HF poses significant challenges in the realm of pharmacological interventions.***

The majority of GPs indicated that they employ stepwise guidelines for pharmacological management.

*“...that’s basically when I look at the guidelines.”* (GP 05, with 3 years of experience).

The Chinese guidelines for managing CPSP are beneficial because of their systematic approaches. However, GPs encounter challenges in selecting optimal medication because of the extensive range of options within each category, such as nonsteroidal anti-inflammatory drugs (NSAIDs), central analgesics, antiepileptics, and antidepressants.

*“I do not comprehend them. There are a great deal of them. I am unaware of the distinction between them.”* (GP 11, with 11 years of experience).

Therefore, GPs are restricted to prescribing well-known or familiar pharmaceuticals.

*“Very intimidating. Sincerely, I wish that there was just one of each. I suppose we’ll discover our favorites, and we’ll stick with them.”* (GP 15, with 21 years of experience).

The participants further contemplated the difficulties they encountered when selecting the most suitable medication for their patients. Occasionally, the introduction of novel medications by pain specialists without the corresponding elucidation of the underlying rationale left GPs perplexed and confronted with complexity. One GP explained,

*“For instance, upon their return from an evaluation conducted by a pain specialist, it was observed that they were prescribed a medication unfamiliar to me on 50 % of occasions. This complexity perplexes me, as I am uncertain of the efficacy and advantages these drugs possess compared to the ones I am acquainted with, thereby confusing.”* (GP 08, with 15 years of experience).

Furthermore, it was found that GPs encountered difficulties staying abreast of recent advancements in medications and technologies utilized to manage CPSP. According to the interviews, GPs primarily acquired knowledge about medications through advertisements in medical journals or educational initiatives sponsored by pharmaceutical companies. GPs acknowledged the need to enhance their understanding of medication management for the treatment of CPSP after HF. However, they expressed a preference for succinct evidence-based information not influenced by the pharmaceutical industry, specifically regarding medications.

*“Keeping up with the latest analgesic drugs and techniques is unquestionably difficult.”* (GP 17, with 29 years of experience).

*“Initially, I found the registrar training quite overwhelming due to numerous drug representatives showcasing their products alongside extensive charts. Consequently, I encountered difficulty comprehending each product’s relative merits and determining their superiority.”* (GP 01, with 4 years of experience).

***Theme 3. Care optimization within constrained consultation time.***

Follow-up consultations for CPSP after HF are usually limited to a standard consultation, with limitations imposed by the accessibility of GPs. This constraint exhibits variability contingent on the scale and geographical setting of community practices. GPs have identified GP staffing and clinic hours as obstacles that impede the provision of comprehensive reviews and management guidance during standard consultations. For example, one GP stated,

*“Navigating through the substantial workload within a 15-min appointment poses considerable challenges.”* (GP 04, with 5 years of experience).

Patients with CPSP after HF frequently present with comorbidities, necessitating follow-up visits beyond the scope of addressing CPSP alone. These visits also included the management of acute or chronic issues. Participants acknowledged the difficulty of delivering optimal follow-up care within the constraints of the standard consultation duration. GPs prioritize addressing patient needs, values, and expectations as a primary concern. One GP explained that,

*“The scheduling conflicts between patients and doctors frequently arise due to misalignment of their schedules. In this case, the patient presents many issues, while I, as a medical professional, am confronted with a series of CPSP problems requiring attention. Regrettably, it is currently unfeasible for me to address these concerns due to temporal constraints.”* (GP 13, with 25 years of experience).

Numerous GPs expressed their appreciation for the involvement of physician assistants in providing supplementary support for CPSP management of patients undergoing HF surgery. However, they also emphasized the considerable disparity in the responsibilities undertaken by physician assistants across GP practices in Shanghai, China. Primarily, physician assistants were engaged in aiding pain evaluation, with a subset participating in patient psychoeducation and facilitating postoperative rehabilitation exercises. However, the extent of the involvement of physician assistants exhibited substantial heterogeneity.

***Theme 4. The desire for prompt communication among healthcare professionals was expressed, albeit with*** var ***ying experiences.***

The GPs recognized hospital discharge summaries as a crucial means of communication, with the majority expressing satisfaction with the promptness of electronically transmitted summaries. Nevertheless, their sentiments regarding the content were ambivalent, as they exhibited inconsistency based on the workplace and affiliated hospital; only a tiny proportion of GPs reported contentment with the provided information.

*“The significant improvement witnessed in recent years can be attributed to the availability of hospital discharge summaries in our locality. These summaries effectively communicate the follow-up plan to GPs and outline the specific actions they are expected to undertake. This development has proven to be highly advantageous.”* (GP 16, with 26 years of experience).

In contrast, additional GPs expressed significant apprehension regarding the discharge summaries they had received, suggesting that the hospital proactively schedules follow-up appointments before the patient is discharged.

*“There are numerous areas that could benefit from improvement. To be candid, I perceive the medication aspect to be of limited utility. This is particularly evident concerning the events that transpired during the admission process. Additionally, one of my primary sources of dissatisfaction is the lack of thorough follow-up and monitoring of the outcomes.”* (GP 09, with 17 years of experience).

*“It is not customary for the patient to be instructed to schedule an appointment with the GP, and it would be highly beneficial if the hospital personnel could assist the patient in arranging a subsequent appointment with the GP before their discharge, as it promotes effective continuity of care.”* (GP 10, with 10 years of experience).

During the interviews, GPs expressed their concerns regarding the challenges they face when communicating with outpatient hospital specialists in superior hospitals.

*“The hospital specialists occasionally modify and alter treatment plans without consulting the community team or general practitioner, resulting in a lack of communication and understanding regarding the rationale behind medication choices. This situation can be quite exasperating.”* (GP 14, with 11 years of experience).

Healthcare providers frequently possess disciplinary perspectives on patient requirements and approaches to care. The GPs emphasized the significance of interprofessional collaboration rather than individualistic practices. Respondents preferred receiving comprehensive feedback from other healthcare professionals involved in patient care as this can contribute to the provision of enhanced personalized CPSP management for patients following HF surgery.

*“The communication from a rehabilitation clinic specializing in orthopaedic or geriatric rehabilitation provides a concise overview yet lacks direct personalization towards the individual. I strongly advocate for the implementation of an individualized approach, as it has the potential to impact outcomes significantly.”* (GP 16, with 26 years of experience).

***Theme 5. Limited access to additional healthcare services hindered the GPs’ ability to deliver optimal care.***

GPs voiced frustration regarding their inability to deliver optimal care for patients suffering from CPSP, while expressing concerns about the accessibility of referral services. Barriers to interdisciplinary pain treatment were identified, including the lower priority given to GP referrals in the public sector, the financial implications associated with private programs, and the limited availability of such programs.

*“The cost of private allied health services is exorbitant, rendering it unattainable for my patients, despite their participation in team care arrangements and efforts to mitigate substantial medical expenses.”* (GP 10, with 10 years of experience).

The participants expressed their belief that numerous factors could be effectively addressed; notably, they opined that hospital staff should take responsibility for organizing referrals for patients after HF surgery before discharge.

Most GPs expressed challenges in accessing outpatient pain specialists when necessary. These difficulties stemmed from issues of accessibility and affordability, which were attributed to the limited availability of pain specialist physicians, extended waiting periods in both the public and private sectors, and the associated costs.

*“The waiting period to consult a specialist in this area is considerably lengthy due to the limited availability of one or two private pain specialist physicians. Given our disadvantaged socioeconomic status, most individuals cannot afford private consultations. Consequently, accessing specialist care in public hospitals also entails enduring extended waiting periods.”* (GP 09, with 17 years of experience).

***Theme 6. Patient adherence to CPSP management.***

GPs experienced a sense of responsibility in motivating patients to discontinue long-term opiate use, yet encountered difficulties when patients continued to exhibit opioid dependence despite receiving support to quit. The GPs acknowledged that the ultimate responsibility for their patients’ health rested with them. Moreover, the participants highlighted issues arising from the lack of continuity in patient care, as patients often sought treatment from multiple GPs without registering with a single practice in Shanghai. This disruption in information and management continuity was perceived to have a detrimental effect on the delivery of optimal care.

*“It is frustrating when the patients have to visit one doctor for a specific issue and then seek assistance from another doctor for a different concern.”* (GP 06, with 12 years of experience).

*“Obtaining information can be challenging, particularly when conveying it to patients who consult multiple physicians. Acknowledging that each doctor may offer distinct perspectives, each possessing its own merits, is crucial. Consequently, it is imperative to establish a relationship with a single healthcare provider whom one finds agreeable and trustworthy.”* (GP 12, with 5 years of experience).

## 4 Discussion

**Figure 1:**
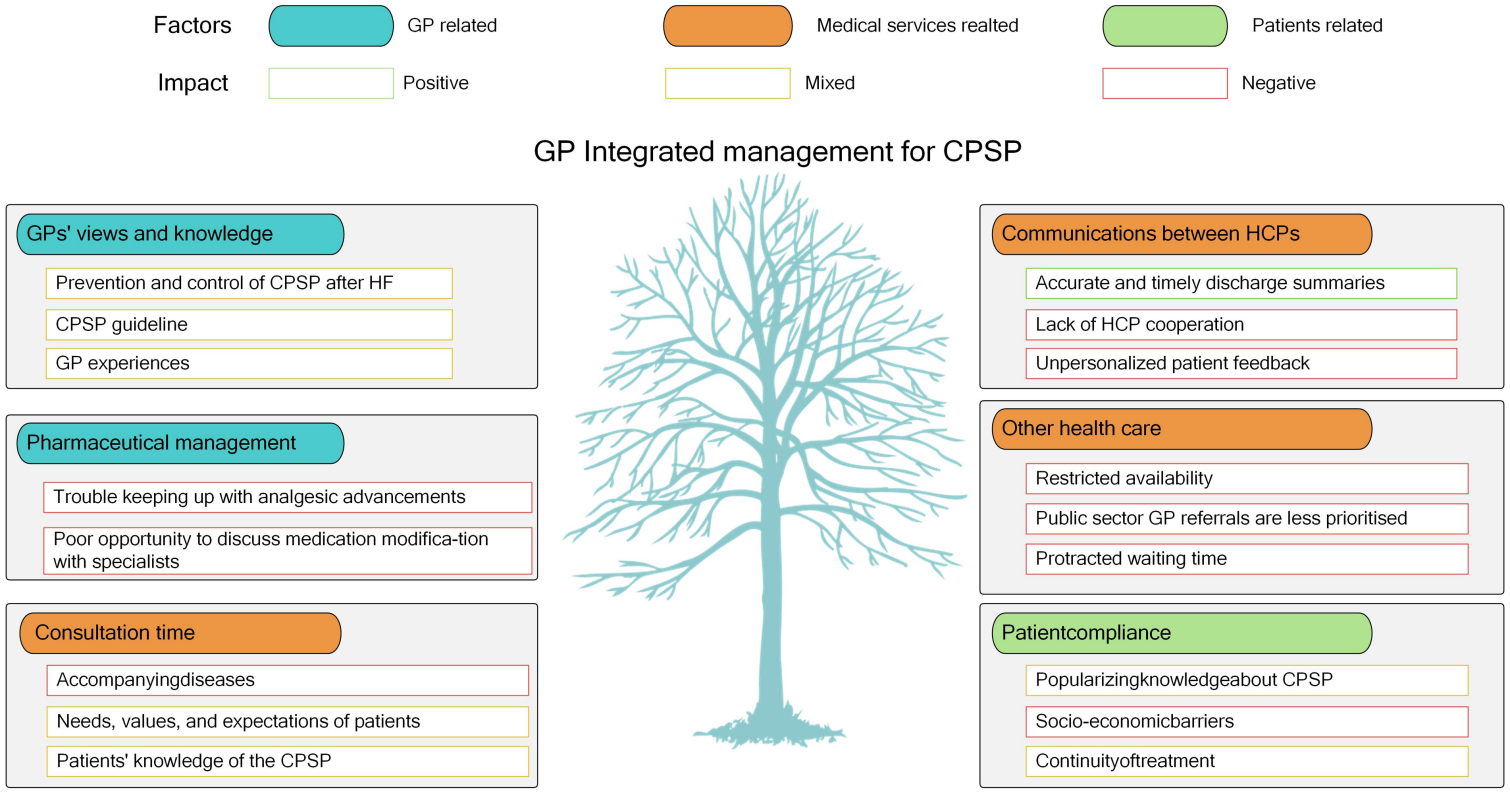
Factors impacting GP comprehensive care of CPSP patients following HF. CPSP, chronic postsurgical pain; HF, hip fracture.

This study offers comprehensive insights into the experiences of GPs responsible for managing patients with CPSP after HF in Shanghai, China. The provision of post-exacerbation care is influenced by various factors including GP knowledge, variations in care based on their perceived priorities, consultation time constraints, challenges in pharmacological management, communication issues among healthcare professionals, patient compliance, and difficulties in accessing healthcare services (Figure 1). The interviews also provided insights into the primary factors contributing to these issues, including the perspectives of GPs, patients, and health services.

A limited number of participants discussed the potential contribution of general practice in mitigating chronic pain after HF surgery within the primary care environment. The global underutilization of CPSP management in primary care settings can be attributed to various factors, such as multimodal or balanced analgesia, minimally invasive blocking techniques, psychological counseling, self-directed pedaling-based rehabilitation, and personalized, targeted discharge plans (21, 22). The Chinese government has prioritized improving the quality of chronic disease management in primary care in the past decade. Many Chinese hospital associations participated in the Single Disease Quality Management Project, which included postoperative pain and its effective treatment as benchmarks for hospital performance (23). Moreover, access to primary care has improved following initiation of the State Council’s extensive healthcare reforms, leading to the attainment of UHC for healthcare at all levels (24). The majority of case-based payments to GPs and hospital specialists are covered by national medical insurance (25). However, our study identified several barriers, including lengthy wait lists, low acceptance of GP referrals for public programs, financial constraints associated with private programs, and limited accessibility of local programs. This phenomenon could be attributed to a sampling bias toward GPs from higher socioeconomic backgrounds, particularly in Shanghai. However, this may also indicate a limited understanding of the availability of local medical insurance. Further investigation is necessary to explore strategies to address obstacles in implementing CPSP management in primary care settings.

One of the primary issues identified in the interviews was the lack of clarity surrounding the pharmacological treatment of CPSP. Analgesic and psychiatric medication utilization was evaluated as part of an in-home medication review (26). Recent shifts in knowledge regarding the efficacy and safety of opioid medications have contributed to increased uncertainty regarding the management of CPSP (27). GPs expressed the need for more precise guidance on selecting pain therapy at each stage of stepwise management, particularly considering the frequent advancements in clinical trials, technological advancements, and combination therapy alternatives. However, obtaining such information from authoritative sources that are free from industry influence is crucial. Therefore, entities such as the National Prescribing Services should offer insights into the role and appropriateness of National Health Service (NHS)-based community and third-sector services in China.

The significance of teamwork and communication in ensuring effective patient care has been widely acknowledged. Nevertheless, the participants in this study reported infrequent receipt of adequate clinical information from hospitalized patients and limited involvement in shared decision-making with fellow healthcare professionals. Previous research on GPs and specialists has identified common concerns and challenges in communication, indicating the necessity for continuous improvement in the exchange of feedback (28–30). The inclusion of GPs in the discharge-planning process is recommended by the CPSP guidelines. However, according to China’s Quality Health Service Standards, transferring clinical information for community-based care is not currently considered a fundamental requirement for hospital personnel. In Europe, it has been reported that 60% of patients suffering from chronic pain have sought medical assistance between two and nine times within the past 6 months (31). Given the likelihood of more visits for pain management in China, it may be necessary to conduct future audits to accurately assess compliance with these guidelines.

Variability in the clinical priorities of the interviewees was observed, which affected the implementation of the CPSP guidelines. Older adults with chronic pain and multiple chronic conditions pose distinct pain management challenges (32). Similar to previous research, the clinical experience and perception of practitioners were deemed significant, but the needs and expectations of patients were also recognized as influential factors in adherence to clinical practice guidelines (33). The patient-centric nature of Chinese general practice, which emphasizes the importance of patient narratives and shared decision-making, can challenge the provision of evidence-based care (34, 35). Medical practitioners must improve their knowledge and skills. Therefore, exploring strategies that enhance familiarity with the guidelines may be necessary. To effectively promote the adoption of guideline-based care, interventions should be comprehensive and customized to address the specific barriers inherent in the health systems and funding models within which GPs operate (36). Using guidelines in clinical practice is more likely when they are simple, relevant to practice, and perceived to be significant.

It is important to note that this study had some limitations that may affect its interpretation. First, our qualitative research approach facilitated comprehensive data collection from significant perspectives on general practice care for patients with CPSP following HF. Although our sample was self-selected (i.e., convenience sampling), none of the participants indicated a specific inclination toward pain-related ailments. Given the rarity of “special interest pathways” among Chinese GPs in pain management, this outcome was anticipated. Nonetheless, our sample encompassed various GPs with varying attributes, including experience and geographical location, ensuring a diverse representation. Second, including a GP as the interviewer may have enhanced the quality and depth of the interview data. Previous research suggests that professionals engaged in interviews with their peers can elicit insightful and comprehensive responses. However, it is essential to acknowledge the potential influence of the interviewer’s biases and perspectives on the conversation, which may result in a limited understanding of the subject matter, known as “conceptual blindness.” Experienced qualitative researchers oversaw the analysis process to mitigate this risk, ensuring methodological rigor and reflective examination of the data. Third, the analysis followed the 6-step approach to reflexive thematic analysis and was performed by only the primary investigator instead of all investigators, which may have biased the analysis results. Group meeting decision-making may be necessary in future studies.

In conclusion, this study offers significant insights into the provision of GP care to patients with CPSP after HF in China. This emphasizes the importance of GPs staying up-to-date with evidence-based guidelines and suggests additional support is required to ensure that clinical practice is informed by evidence. Furthermore, there is ample room for further exploration of strategies to improve communication between hospitals and primary care settings, such as involving GPs during hospital admission. The potential enhancement of CPSP care can be achieved by implementing disease-specific templated clinical handovers informed by GPs and initiating initiatives to strengthen patient and caregiver engagement in discharge planning.

## Data availability statement

The raw data supporting the conclusions of this article will be made available by the authors, without undue reservation.

## Ethics statement

This study was conducted in accordance with China law no. 2023–04 of February 18, 2023, which falls outside the scope of research involving human subjects. Consequently, it does not necessitate submission to an ethics committee as it primarily constitutes an opinion survey.

## Author contributions

WC: Conceptualization, Data curation, Methodology, Visualization, Writing – original draft. YY: Methodology, Resources, Supervision, Validation, Visualization, Conceptualization, Investigation, Writing – original draft. JY: Investigation, Methodology, Software, Supervision, Writing – original draft. QL: Conceptualization, Formal analysis, Investigation, Software, Supervision, Writing – review & editing. CX: Data curation, Investigation, Resources, Software, Validation, Visualization, Formal analysis, Writing – review & editing.
